# Lessons learned for pandemic preparedness in the neurodegenerative research and clinical fields: an advice report based on Parkinson’s disease as an example

**DOI:** 10.1186/s12883-024-03975-8

**Published:** 2024-12-05

**Authors:** Marije J. Splinter, Emily J. Henderson, Yoav Ben-Shlomo, Sirwan K. L. Darweesh, Pawel Sowa, Frank J. Wolters, Premysl Velek, Hannie J. E. M. Meijerink, Paulus Bakx, M. Arfan Ikram, Evelien I. T. de Schepper, M. Kamran Ikram, Silvan Licher

**Affiliations:** 1 Department of Epidemiology, Erasmus University Medical Centre, Rotterdam, The Netherlands; 2 Department of Population Health Sciences, Bristol Medical School, University of Bristol, Bristol, UK; 3 Royal United Hospital Bath NHS Foundation Trust, Bath, UK; 4 Department of Neurology, Radboud University Medical Centre, Nijmegen, The Netherlands; 5 Department of Population Medicine and Lifestyle Diseases Prevention, Medical University of Bialystok, Bialystok, Poland; 6 Department of Neurology, Erasmus University Medical Centre, Rotterdam, The Netherlands; 7 Department of General Practice, Erasmus University Medical Centre, Rotterdam, The Netherlands; 8 Parkinson Vereniging, Bunnik, The Netherlands

**Keywords:** COVID-19, Pandemic preparedness, Neurodegenerative diseases, Cohort study, Healthcare utilisation

## Abstract

**Background:**

A sustainable pandemic preparedness strategy is essential to ensure equitable access to healthcare for individuals with neurodegenerative diseases. Moreover, it is vital to provide clinicians and researchers in the neurodegenerative disease fields with resources and infrastructure to ensure continuity of their work during a (health) crisis.

**Methods:**

We established an international collaboration between researchers, clinicians, and patient representatives from the Netherlands, Poland, and the United Kingdom. We co-created a pandemic preparedness plan primarily informed by examples from those affected by or working in the field of Parkinson’s disease, with potential application to other neurodegenerative diseases or the general population. This plan builds upon insights and experiences from four population-based studies during the COVID-19 pandemic. Between March and November 2023, we organised two hybrid meetings in Bristol (United Kingdom) and Rotterdam (the Netherlands), and two online meetings.

**Results:**

Research recommendations included three core factors in questionnaire design during health crises: 1) using existing, validated questions, 2) questionnaire adaptability and flexibility, and 3) testing within and outside the research group. Additionally, we addressed burden of participation, and we advocated for robust data sharing practices, underlining the importance of regulatory measures extending beyond the COVID-19 pandemic. We also shared clinical perspectives, including strategies to mitigate social isolation; challenges in virtual versus in-person consultations; and systemic changes to recognise and prevent moral injury in healthcare professionals.

**Conclusion:**

In this pandemic preparedness plan, we provide research and clinical recommendations tailored to the field of Parkinson’s disease, with broader relevance to other neurodegenerative diseases and the general population. This establishes an essential framework for setting up new studies and safeguarding research and clinical practices when a new pandemic or other (health) crisis emerges.

**Graphical Abstract:**

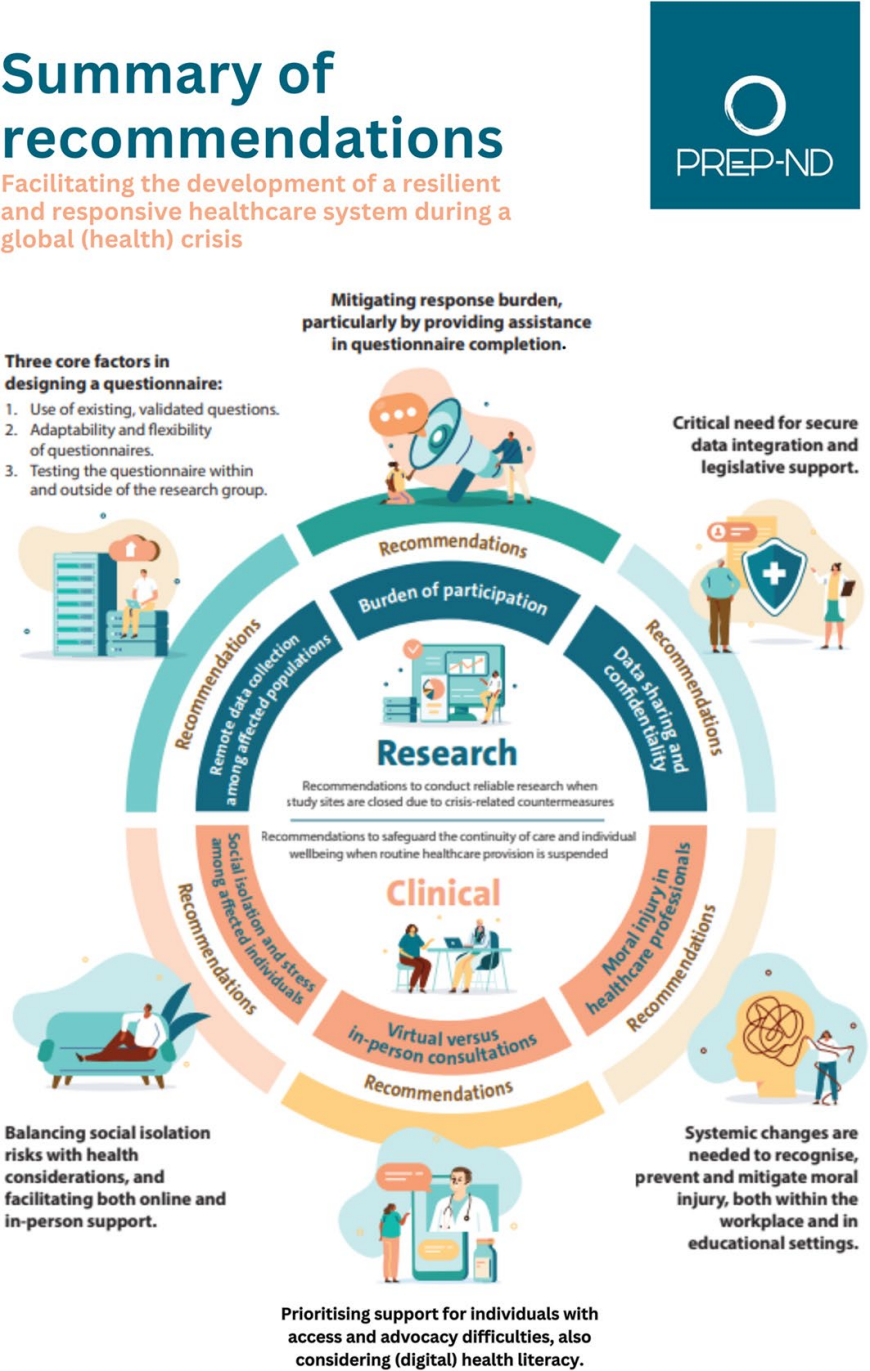

## Keypoints

• A sustainable pandemic preparedness strategy is essential for equitable healthcare access and continuity of research and clinical practice in the neurodegenerative disease fields.

• We established an international collaboration involving researchers, clinicians, and patient representatives from the Netherlands, Poland, and the UK, to co-create a pandemic preparedness plan, drawing upon their experiences in four population-based studies during the COVID-19 pandemic.

• Research recommendations included key factors in remote data collection, strategies to reduce the burden of participation, and considerations in terms of data sharing and confidentiality.

• Clinical strategies highlighted mitigating social isolation, challenges in virtual consultations, and preventing moral injury in healthcare professionals.

## Introduction

The COVID-19 pandemic has led to suboptimal care for individuals at risk of or living with neurodegenerative diseases [1, 2]. There were notable declines in the incidence of registered dementia and all-cause parkinsonism diagnoses, as well as related hospital admissions, compared to pre-pandemic levels [3–8]. Changes in healthcare-seeking behaviour may be underlying these declines, resulting from limited access to healthcare due to deferral of elective and non-urgent consultations [9–13]. Furthermore, the rapid implementation of telemedicine throughout the entire healthcare system proved to be challenging, especially in adapting to the needs of individuals with cognitive impairments or sensory issues [14, 15]. These developments might have exacerbated pre-existing inequalities in health outcomes, as the most vulnerable individuals were most affected, with a synergistic interplay of age, socioeconomic status, and social deprivation [16, 17].

The expected seasonal recurrence of (new variants of) COVID-19 and the potential emergence of other airborne infectious diseases underscore the need for a sustainable healthcare preparedness strategy [18, 19]. This is particularly essential to ensure equitable access to care for individuals with chronic diseases. Moreover, it is vital to provide clinicians and researchers in the field of neurodegenerative diseases with resources and infrastructure to ensure continuity of their work during a pandemic or other (inter)national (health) crisis.

To address this need, we established an international collaboration consisting of experts with complementary backgrounds, including researchers with a clinical background and those in the fields of epidemiology and public health. Collectively, we constructed a pandemic preparedness advice report through co-creation with patients’ and caregivers’ representatives of the Dutch Parkinson’s Association, with the aim of redefining lessons learned regarding healthcare provision, healthcare utilisation, and research into strategies and practices that could be applied during future pandemics. For scientists, we provide recommendations on conducting reliable research when study sites are closed due to pandemic-related countermeasures. For patients and clinicians, we present guidelines to safeguard the continuity of care and individual wellbeing when routine healthcare provision is suspended.

## Methods

### Context of the advice report: the COVID-19 pandemic

In December 2019, an outbreak of SARS-CoV-2 was registered in the Chinese municipality of Wuhan. In the following months, the respiratory virus had spread mostly within China but also to 28 additional countries [20]. In the European region, Italy was the first country to implement a nationwide quarantine, after which other countries followed. Nearly all gatherings and events were prohibited, international travel was either cancelled or restricted, non-essential retail stores were closed, and both educational and work environments primarily operated online. These countermeasures were continuously evaluated and subsequently relaxed or tightened based on infection and hospitalisation rates. However, fundamental hygiene practices, such as hand washing, wearing face masks in public, and avoiding in-person contact when experiencing COVID-19 symptoms, remained in place [21]. As of December 2023, the European region had reported more than 277 million confirmed cases and 2.2 million deaths attributable to COVID-19 [22].

### Selection of participants

PREP-ND is an international collaboration consisting of a total of thirteen members: eleven with a research and/or clinical background, along with two patient representatives from the Netherlands, Poland, and the United Kingdom. All participants were invited via email.

For the recruitment of researchers and clinicians, we employed purposeful sampling, which is a qualitative, nonprobability sampling technique that involves identifying individuals that are especially knowledgeable about or experienced with the phenomenon of interest [23]. In the context of this report, the selected participants have extensive experience in collecting population-level and clinical data in cohort studies and/or with providing care during the COVID-19 pandemic, particularly within the field of Parkinson’s disease and the general population. Their academic backgrounds included epidemiology, general practice, neurology, public health, sociology, and statistics, ensuring an interdisciplinary perspective on (health) crisis preparedness.

We recruited the patient representatives through snowball sampling, a convenience sampling method [24]. These representatives were enlisted from the established network of one of the already involved participants, with whom they had previously collaborated on other projects.

### Development of recommendations

Over the course of twelve months, we organised two hybrid meetings, one in Bristol (United Kingdom, 15 March 2023) and another in Rotterdam (The Netherlands, 26 June 2023), and two online meetings.

During the first two-hour hybrid meeting, attendees prepared an overview of their experiences with healthcare provision, access to healthcare, and/or conducting research during the COVID-19 pandemic according to a pre-specified topic list (Table 1). The aim of this meeting was to identify best practices and lessons learned, which we could collectively transform into recommendations for a future (health) crisis. We shared and explored the experiences in a round-table discussion, delving into the social, cultural and political differences between our healthcare systems during the pandemic. Attendees also had the opportunity to propose and discuss additional topics that they considered particularly relevant. The meeting was recorded, transcribed and summarised by one PREP-ND member with a background in qualitative research methods. This summary distinguished between research and clinical perspectives, classifying all discussed insights and perspectives into candidate recommendations with corresponding sub-themes [25].

**Table 1:** Topic list

Subject	Attendee	Questions
Impact of COVID-19	Healthcare provider/researcher	• How did the COVID-19 pandemic impact your daily work as a healthcare provider/researcher?
	Patient representative	• How did the COVID-19 pandemic impact your daily life?
Challenges in providing/accessing healthcare	Healthcare provider/researcher	• Can you describe any particularly difficult or challenging situations that you faced while providing care/conducting research during the COVID-19 pandemic? • Did you overcome these challenges, and how did you do that?
	Patient representative	• Can you describe any particularly difficult or challenging situations that you faced while accessing care during the COVID-19 pandemic? • Did you overcome these challenges, and how did you do that?
Adaptation and response strategies	Healthcare provider	• What measures did you and/or your organisation take to ensure that patients could continue to receive care during the COVID-19 pandemic? • What measures did you and/or your organisation take to ensure that those who were unable to come to the hospital or clinic in-person could continue to receive care during the COVID-19 pandemic?
	Researcher	• What measures did you and/or your organisation take to ensure continuity of research during the COVID-19 pandemic?
	Patient representative	• What measures did your healthcare provider take to ensure that you could continue to receive care during the COVID-19 pandemic? • What measures did your healthcare provider take to ensure that those who were unable to come to the hospital or clinic in-person could continue to receive care during the COVID-19 pandemic?
Lessons learned	Healthcare provider/researcher/patient representative	• Looking back on the past three years of COVID-19, what lessons have you learned about healthcare delivery and access to care during a health crisis? • How will you apply these lessons in the future?

In the second two-hour hybrid consensus meeting, we reviewed these recommendations with group members who were unable to attend the first meeting, maintaining flexibility to revisit and refine (sub-) themes, in line with recommendations by Braun and Clarke [25]. Employing an iterative approach, we agreed on the overall structure of the final advice report, which we drafted over the consecutive months. We evaluated and revised its content in the two remaining online meetings, ensuring thorough review of the final report by all members.

### COVID-19 (sub-)studies

The recommendations provided in this advice report are based on PREP-ND members’ involvement in either the following (cohort) studies, ranging from population-based to clinical settings, or a patient association (Table 2).

**Table 2:** Referenced cohort studies and patient association

Study/association	Description
The Rotterdam Study [26, 27]	From April 2020 to December 2021, seven questionnaires were distributed to community-dwelling participants of the ongoing population-based Rotterdam Study in the district Ommoord in Rotterdam, the Netherlands. These questionnaires covered a range of topics, including COVID-19-related symptoms, socioeconomic factors, lifestyle, and healthcare utilisation. The frequency of the questionnaires was based on the COVID-19 infection curves in the Netherlands
PRIME-XS [20]	This single-centre, cross-sectional study began in September 2020 and involved individuals with parkinsonism and their primary informal caregivers in the catchment area of Royal United Hospital Bath NHS Foundation Trust in the United Kingdom. Participants were provided with a questionnaire that could be completed by the individuals with parkinsonism themselves, their caregivers, or a research assistant over the phone. The survey included questions on COVID-19-related symptoms, healthcare utilisation, sociodemographic factors, medication use, and PD-specific measures
PRIME-NL [28, 29]	This prospective, observational study is ongoing from January 2020 through December 2025 and includes individuals diagnosed with Parkinson’s disease or atypical parkinsonism who are receiving treatment in one of the four community hospitals that form the PRIME Parkinson care region of the Netherlands. The study aims to analyse variations in care and their impact on perceived quality of life by collecting data from healthcare claims and annual questionnaires completed by persons with parkinsonism, their caregivers, and healthcare providers
Bialystok PLUS [13, 30–32]	The research project ‘Rise or fall? Short- and long-term health and psychosocial trajectories of the COVID-19 pandemic’ was embedded within the Bialystok PLUS cohort study. It examines the short- and long-term health and psychosocial trajectories in the general population during the COVID-19 pandemic. It includes research centre visits by post-COVID-19 individuals, a general population survey using a CAWI questionnaire, and the analysis of the impact of the pandemic on mental health as part of the COH-FIT project
The Parkinson’s Association [33]	The Dutch Parkinson’s Association represents individuals with Parkinson’s disease or parkinsonism, and their loved ones. This association organises meetings and courses, provides up-to-date and objective information, and is a critical advocate in political and media settings. It also safeguards quality of care and ensures the voice of those involved with Parkinson’s disease or parkinsonism is heard in scientific research. Both patient representatives involved in PREP-ND were affiliated with this association

## Results

The four meetings revealed the following recommendations with corresponding sub-themes for both the continuity of research and for the improvement of clinical practice and individual wellbeing during a (health) crisis.

### Recommendations to facilitate the continuity of research during a global (health) crisis

#### Theme 1: Remote data collection among affected populations

Nationwide lockdowns and other preventive measures throughout the COVID-19 pandemic caused substantial disruptions to in-person data collection. In response, the researchers involved in PREP-ND established a COVID-19 (sub-)study to ensure research continuity and to gain insights into various aspects of COVID-19, including risk factors, mental and physical health, and healthcare-seeking behaviour and utilisation [13, 27, 28, 34]. This was primarily done by distributing questionnaires that participants could complete from the safety of their home environment. Study protocols of these questionnaires had to be created within a short timeframe due to the need to adapt to the continuously evolving infection rates, leaving limited opportunities for evaluation and adjustment of study design. Summarising the most important lessons learned from this period, we recommend focusing on three core factors in the design of questionnaires during a pandemic:

### Core factor 1: Use of existing, validated questions

There is a need for a framework or standardised questionnaire that is specifically tailored to address the impact of a pandemic on vulnerable individuals. Such questionnaires should have the flexibility for adaptation while allowing for cross-validation and comparison across (cohort) studies. It should also contain suggestions for questions about time-varying factors, such as mood or healthcare utilisation. These questionnaires save valuable time in the process of study design, while still providing high-quality and relevant data. Successful examples of existing, open-access questionnaire databases are the European Social Survey [35], the Joint Research Centre COVID-19 Survey of the European Commission [36], and the International Social Survey Program [37], based on which the questionnaires in the Bialystok PLUS study were developed [13].

### Core factor 2: Adaptability and flexibility of questionnaires

The applicability of an existing questionnaire should be reviewed in light of any specific pandemic-related factors. It is essential to assess whether both the questions and the response options align with the current situation, or if any revisions are necessary. For example, it should be evaluated whether response categories offer sufficient variation in order to minimise the risk of misclassification. Binary response options do not always adequately capture the complexity of participants’ experiences. To illustrate this notion, the following question that was used by the COVID-19 sub-study within the Rotterdam Study to inquire about healthcare avoidance: “Did you have symptoms for which you did not contact your general practitioner or medical specialist because of the COVID-19 pandemic” to which respondents could either answer yes or no. However, those who answered ‘no’ were not necessarily non-avoiders, as among them could also have been individuals who did not experience any symptoms and, therefore, were not at risk of avoiding healthcare. Hence, the question should have incorporated additional response options or included the possibility for participants to provide a free-text comment (Table 3). The latter option ensures that the entire spectrum of viewpoints is captured in the question. Nevertheless, researchers should evaluate the feasibility of performing additional data cleaning tasks that are associated with free-text responses [38]. Furthermore, it is important to consider the order in which responses are presented, taking into account the risk of acquiescence. This bias arises when the first response options that are listed are typically affirmative or the most socially acceptable, potentially leading to a perception that these are the preferred choices [39].

**Table 3:** Example question

Former version	Recommended version
1. Did you have symptoms for which you did not contact your GP or medical specialist because of the COVID-19 pandemic? • No • Yes	1. Did you have symptoms for which you did not contact your GP or medical specialist since (date)? • I did not have any symptoms (continue to question 5) • I had symptoms, but I contacted my GP or medical specialist for them (continue to question 4) • I had symptoms for which I did not contact my GP or medical specialist (continue to question 2)
	2. Which symptoms did you have since (date) for which you did not contact your GP or medical specialist?
	• List with pre-specified symptoms, and a free-text option
	3. What was the most important reason for not contacting your GP or medical specialist?
	• Fear of becoming infected with COVID-19 • Fear of burdening my healthcare provider • I did not expect to get access to care (for example, I thought there would not be a hospital bed for me) • Financial reasons • Other… (free-text)
	4. Did your symptoms, to your own insight, require direct medical attention?
	• None of my symptoms required direct medical attention • At least one of my symptoms required direct medical attention, and I also received that • At least one of my symptoms required direct medical attention, but I did not (directly) receive that. I experienced this once or twice • At least one of my symptoms required direct medical attention, but I did not (directly) receive that. I experienced this 3, 4 or 5 times • At least one of my symptoms required direct medical attention, but I did not (directly) receive that. I experienced this 6 or more times

### Core factor 3: Testing the questionnaire within and outside of the research group

Questionnaires developed during the COVID-19 pandemic mainly reflected the perspective of the research group itself, as the rapid development of the virus necessitated quick establishment of new research initiatives, limiting the ability to incorporate a broader range of perspectives. However, this approach may not accurately capture the experiences of the target population. To mitigate potential bias, we recommend establishing a permanent patient or participant panel that plays a pivotal role in both study design and data collection. This becomes imperative in cases where existing questionnaires are not fully applicable to a specific health crisis, necessitating adjustments or the introduction of new questions.

Prior to distributing the questionnaire, an obvious yet often overlooked step during the acute phase of a pandemic relates to the rigorous testing of the questionnaire by both this patient/participant panel and the research group. The primary responsibility of the research group is to evaluate the structure and flow of the questionnaire, particularly for questionnaires that contain skip questions. This is vital to provide a seamless experience for participants and to prevent them from being directed to sections they are not supposed to complete. The patient/participant panel, on the other hand, should review the content of the questionnaire to ensure it encompasses a wide range of common perspectives, experiences and whether all answer options are captured sufficiently. Partly based on the target audience, the research group and patient/participant panel should decide on the distribution format of the survey, whether on paper, digitally, or both. Although paper questionnaires are typically more costly and have a higher data cleaning burden, a combination of both paper and digital methods is assumed to enhance response rates and improve generalisability, as this approach enables inclusion of individuals without computer access or with limited digital skills [40]. It can also be considered to include a question about the participant’s preferred format for follow-up questionnaires.

#### Theme 2: Burden of participation

It is suggested that questionnaire length and cognitive load can increase the burden of participation in survey research [41]. These factors may result in lower response rates and reduced questionnaire completion. For this reason, researchers often decide to refrain from distributing overly lengthy questionnaires. While being mindful of questionnaire length, we recommend considering additional ways to minimise response burden and enhance response rates, for example by providing assistance in completing the questionnaire. This method was exemplified within the PRIME-XS study, which involved a vulnerable population of individuals with parkinsonism and primary informal caregivers of individuals with parkinsonism. Those who did not respond to the initial invitation letter received one or two telephone calls from the study team, which served the purpose of answering questions, inquiring about the need for support in participation, identifying potential language barriers, or assessing the capacity of the individual to provide informed consent [34]. In cases where individuals were unable to consent, a close friend or relative was allowed to act as a personal consultee. Moreover, participants were not obliged to complete the questionnaire in one sitting, but had the flexibility to spread it over several days, allowing for a more accommodating, participant-centred approach [34]. The key principle underlying these efforts was to convey genuine appreciation for participants’ involvement, emphasising their freedom to withdraw their participation at any time for any reason. In this way, vulnerable individuals were not immediately excluded from participation but received additional assistance to enable their engagement.

#### Theme 3: Data sharing and confidentiality

Research during the COVID-19 pandemic generated a large amount of available data, but also created challenges in effectively connecting multiple data sources for long-term use. The urgency of the crisis necessitated an override of the usual protocols, facilitated largely due to temporary regulatory measures, yet this approach fell short of establishing a lasting framework for integrating data sources. Regulatory measures are needed beyond the duration of the pandemic, which carefully balance privacy regulations with the benefit of health research for public health. Informed consent forms that accommodate for data sharing, and universal templates for data sharing agreements may further enhance rapid, joint research efforts during pandemics. Moreover, an established and continuously-funded research data sharing platform is needed, where researchers can securely upload their data to a repository that already has all requisite permissions in place [42]. These efforts hinge on involvement of governmental agencies and implementation of supporting, permanent legislation. A successful initiative is the UK Longitudinal Linkage Collaboration, which combines data from major interdisciplinary and pan-UK Longitudinal Population Studies (LPS) with COVID-19-related records. All data is first processed by the original study and the National Health Service (NHS) to remove identifiers such as name and address. Then, the data is stored and analysed in a secure research computer, from which no data can be removed. This setup facilitates pooled analyses within a functionally anonymous Trusted Research Environment (TRE), regulated under the Digital Economy Act[43]. Examples of data suitable for these research environments are the Microdata from Statistics Netherlands, comprising linkable, anonymised data that can be accessed through a secure environment known as the Remote Access (RA) environment. Similarly, the Dutch National Institute for Public Health and the Environment (RIVM) collected individual-level data during the COVID-19 pandemic such as vaccination status and COVID-19 testing information.

### Recommendations to improve clinical practice and individual wellbeing during a global (health) crisis

#### Theme 1: Social isolation and stress among affected individuals

Being largely confined to the home or usual residence during the COVID-19 pandemic was detrimental for both physical and mental health of individuals with neurodegenerative diseases [44]. Environmental changes during the pandemic affected their cognitive, behavioural, and psychological wellbeing [45, 46]. For instance, individuals with Parkinson’s disease and their caregivers shared that the lack of regular exercise and social interaction resulted in elevated feelings of stress. In-person movement groups that used to take place during the week were cancelled, depriving these individuals of the opportunity to undertake physical activity within settings such as gyms or swimming pools. This was particularly challenging for those without adequate space or equipment in their home environment to exercise. Moreover, the lack of social engagement led to increased feelings of isolation. Patient representatives noted that group exercise can offer a substantial motivational boost that is challenging to replicate when exercising alone. Therefore, we propose carefully balancing the harms of social isolation with the health risks posed by the crisis. In-person meetings for affected individuals with a low risk of severe infection may be facilitated, provided that appropriate protective measures are in place. Individuals who are classified as high-risk should be targeted for online support groups as a safer alternative, which was put into practice by the clinicians affiliated with the PRIME-XS study. These support groups encompassed online patient gatherings, virtual sessions led by a physiotherapist, and employment of social workers to assist those struggling with social isolation. These meetings could also serve as information platforms, providing regular updates and recommendations to affected individuals about pandemic-related factors, such as infection prevention and potential treatment options. Collaboration with disease charities, which have extensive patient networks, is crucial in this approach.

#### Theme 2: Virtual versus in-person consultations

Preserving patient confidentiality presents a challenge in virtual healthcare provision. For instance, conducting private conversations is more feasible when patients reside in larger homes with multiple rooms compared to those in smaller shared apartments. Yet, evaluating patients in their own environment during virtual home visits also offers unique advantages. It could provide insights into their gait and activities of daily living that might not be as apparent in a clinical setting, enabling healthcare providers to make decisions such as repositioning furniture to prevent freezing among individuals with parkinsonism.

In the implementation of telemedicine, it is essential to prioritise the support of individuals who may encounter difficulty in advocating for themselves, considering not just their physical health but also their social circumstances, such as stigma or lack of privacy. These individuals might not feel secure expressing themselves via phone or video calls. Moreover, within the European Union, between one-third and nearly half of the population has low health literacy and almost half lack basic digital skills [47, 48]. To have basic digital skills, individuals must know how to do at least one activity associated with each of the following domains: information and data literacy, communication and collaboration, digital content creation, safety, and problem-solving [47]. Therefore, when deciding who should be targeted with digital consultations, it is vital to account for this digital divide and its impact on different patient groups. Yet, we should refrain from labelling patients as digitally challenged solely based on factors such as their educational attainment, occupation, or ethnic background, as this may contribute to stigmatisation of these groups and unfairly defining them as unskilled or incapable. Instead, the decision to schedule a virtual or in-person consultation should be made in careful collaboration with the patient, taking into account their complex care needs, such as the presence of somatic and/or language barriers that could hinder virtual communication, as well as personal preferences [14].

During a pandemic, a risk stratification could serve as an initial step in this decision, however, a comprehensive understanding of risk necessitates the convergence of various domains, such as environmental factors (aimed at minimising the risk of cross-contamination), patient-specific factors (focused on ensuring patient safety), and healthcare professional factors (regarding their safety and capacity). For individuals with or at risk of neurodegenerative diseases, this multifaceted approach should extend beyond physical symptoms to encompass neuropsychiatric symptoms as well. Affected individuals who strongly prefer in-person consultations despite risk factors should have their preferences carefully considered, but in situations where safety dictates otherwise, physicians may need to overrule these preferences in order to safeguard both healthcare providers and other patients. In such cases, home visits may represent a viable compromise.

#### Theme 3: Moral injury in healthcare professionals

The concept of moral injury has mainly been acknowledged within the field of military psychiatry [49]. However, it has become increasingly recognised as a potential consequence of having delivered healthcare during the COVID-19 pandemic. This condition is characterised by a profound psychological trauma resulting from situations that challenge an individual’s deeply-held moral convictions. It manifests as emotions like guilt, shame, anger, and a sense of alienation from one’s personal identity. Distinguishing moral injury from other related conditions such as moral stress, burnout, and posttraumatic stress disorder (PTSD) is essential for understanding and addressing the unique challenges that healthcare professionals face [50]. Unlike moral stress, moral injury is a persistent and enduring condition that emerged from prolonged exposure to moral conflict. However, moral stress can evolve into moral injury if left unaddressed. In contrast to PTSD, moral injury does not feature symptoms related to fear, and is not contingent upon direct personal harm or threat.

The COVID-19 pandemic has brought attention to the risk of moral injury among healthcare professionals. Providing care during the pandemic was a complex experience for them: many felt a sense of moral conflict regarding the patients they generally treated, but were no longer able to see in-person. Simultaneously, they felt a sense of urgency and duty in delivering the acute care of patients with COVID-19. Moreover, they were concerned with safeguarding their own well-being and the health of those in their personal lives. Other examples of moral injury include the necessity to limit patients’ in-person interactions and separating them from their relatives, as well as feeling unable to provide the level of care that they believed their patients deserved [51].

To prevent or address moral injury, systemic changes are imperative. This begins with healthcare organisations recognising the importance of preventing or reducing moral injury, similar to how burnout reduction is prioritised at institutions like the Mayo Clinic, NHS Lothian/Scotland, and Stanford University [52]. Subsequently, development of infrastructural support systems is essential to facilitate psychological well-being to buffer and mitigate the risk of moral injury in times of crises. This includes evidence-based training programs aimed at recognising and addressing moral injury, ensuring access to mental health support and other counselling services, and fostering a workplace culture that promotes open communication and peer support [50, 53]. Additionally, in anticipation of potential future health crises resembling the COVID-19 pandemic, educational institutions training healthcare professionals should integrate moral injury awareness and resilience-building techniques into their curricula to better equip them for the moral challenges they may face in their careers.

## Summary and conclusions

For this advice report, we brought together a group of experts to develop recommendations for the continuity of research and healthcare in the field of neurodegenerative diseases during a global (health) crisis. Research recommendations included key factors in remote data collection; strategies to reduce the burden of participation; and considerations in terms of data sharing and confidentiality. From a clinical perspective, we emphasised the importance of mitigating social isolation and stress among affected individuals; we shared guidelines in the use of virtual and in-person consultations; and we stressed the need to recognise and address moral injury in healthcare professionals.

The major strength of this report is the collaboration among a diverse group of contributors, bringing together scientific, clinical, and patient perspectives. Yet, as we emphasised earlier, each (health) crisis may require specific adaptations of research and clinical practices to effectively protect vulnerable populations. The patient representatives, researchers, and clinicians involved in this advice report were mainly experienced in the field of Parkinson’s disease. As such, the clinical recommendations in this report may not fully address the needs of individuals with other neurodegenerative diseases, such as dementia, where cognitive impairment introduces unique challenges that were not the primary focus of this report. Therefore, future studies should expand to include patients (or their representatives), researchers and clinicians from a wider range of neurodegenerative conditions.

This report will be useful to researchers, clinicians, and policymakers to enhance preparedness for future (health) crises. It is of particular relevance to the field of Parkinson’s disease, including affected individuals, healthcare professionals, and associated research fields. Importantly, this report and existing literature underscore the need to proactively establish pandemic preparedness now, rather than adopting a passive approach until such crises arise [18, 54–56]. These preparedness frameworks should be interdisciplinary and intersectional in scope, extending beyond identifying and monitoring disease outbreaks, and fostering collaboration across the entire scientific spectrum along with patient and public involvement [16, 54, 57–59]. This approach facilitates the development of a resilient and responsive healthcare system that is capable of shielding vulnerable populations from widening healthcare disparities during future crises.

## Data Availability

No datasets were generated or analysed during the current study.
